# Schisantherin A inhibits cell proliferation by regulating glucose metabolism pathway in hepatocellular carcinoma

**DOI:** 10.3389/fphar.2022.1019486

**Published:** 2022-11-08

**Authors:** Fan Feng, Lianhong Pan, Jiaqin Wu, Mingying Liu, Long He, Li Yang, Wei Zhou

**Affiliations:** ^1^ National Innovation and Attracting Talents “111” Base, Key Laboratory of Biorheological Science and Technology, College of Bioengineering, Ministry of Education, Chongqing University, Chongqing, China; ^2^ Chongqing Engineering Research Center of Antitumor Natural Drugs, Chongqing Three Gorges Medical College, Chongqing, China; ^3^ School of Comprehensive Health Management, XiHua University, Chengdu, Sichuan, China; ^4^ School of Artificial Intelligence, Chongqing University of Education, Chongqing, China; ^5^ Chongqing Key Laboratory of Translational Research for Cancer Metastasis and Individualized Treatment, Chongqing University Cancer Hospital, Chongqing, China

**Keywords:** schisantherin A, hepatocellular carcinoma, fructose and pentose phosphate metabolism, galactose metabolism, traditional Chinese medicine

## Abstract

Schisantherin A (STA) is a traditional Chinese medicine extracted from the plant *Schisandra chinensis*, which has a wide range of anti-inflammatory, antioxidant, and other pharmacological effects. This study investigates the anti-hepatocellular carcinoma effects of STA and the underlying mechanisms. STA significantly inhibits the proliferation and migration of Hep3B and HCCLM3 cells *in vitro* in a concentration-dependent manner. RNA-sequencing showed that 77 genes are upregulated and 136 genes are downregulated in STA-treated cells compared with untreated cells. KEGG pathway analysis showed significant enrichment in galactose metabolism as well as in fructose and mannose metabolism. Further gas chromatography-mass spectrometric analysis (GC-MS) confirmed this, indicating that STA significantly inhibits the glucose metabolism pathway of Hep3B cells. Tumor xenograft in nude mice showed that STA has a significant inhibitory effect on tumor growth *in vivo*. In conclusion, our results indicate that STA can inhibit cell proliferation by regulating glucose metabolism, with subsequent anti-tumor effects, and has the potential to be a candidate drug for the treatment of liver cancer.

## 1 Introduction

Liver cancer is an aggressive malignancy with very high morbidity and mortality (Foerster et al., 2022; Lu et al., 2022). The latest data from the Chinese National Cancer Center indicated that the incidence of liver cancer in China ranks fifth, and the mortality rate ranks second (Sung et al., 2021). Primary liver cancer involves three different pathological types of tumors, namely hepatocellular carcinoma (HCC), intrahepatic bile duct carcinoma, and mixed carcinoma. HCC accounts for more than 85%–90% of cases, with a high degree of malignancy, and is prone to recurrence and metastasis (Pan et al., 2021; Khatib et al., 2022). Traditional chemotherapy as a common treatment option can lead to cure and life prolongation for patients with HCC, yet the prognosis for most patients is dire or poor (Fu et al., 2022; Han et al., 2022). Therefore, the quest for potent new drugs for the treatment of HCC is paramount.

Cancer, including HCC, has recently been reconsidered as a metabolic disease, involving changes in the way energy is produced and utilized (Seyfried et al., 2014; Liao et al., 2022). Excessive proliferation of cancer cells requires a large amount of energy and nutrients, resulting in metabolic changes. Studies by Warburg and colleagues in the 1920s showed that tumor cells preferred increasing their energy supply by glycolysis, rather than mitochondrial oxidative phosphorylation (OXPHOS), even under oxygen-rich conditions (Koppenol et al., 2011). This metabolic reprogramming is what distinguishes malignant tumors from normal tissue and is known as the Warburg effect or aerobic glycolysis (Sun et al., 2022). Glycolysis produces ATP very fast, 100 times faster than OXPHOS (Locasale and Cantley, 2010). Tumor cells use glycolysis to accelerate energy acquisition through metabolic reprogramming to support the rapid proliferation of tumor cells (Yang et al., 2022).

Glucose metabolism in HCC cells is characterized by increased glucose uptake and accelerated glycolysis (Li et al., 2017). The acceleration of glycolysis in HCC depends on a number of key rate-limiting enzymes. The first step in glycolysis is the transport of glucose into cells through glucose transporters (GLUTs). Compared with normal hepatocytes, the expression and activity of GLUTs are upregulated in HCC cells (Xia et al., 2020). Wang et al. found that the highly expressed proto-oncogene MYC in HCC could accelerate glycolysis in HCC cells through the regulation of lactic dehydrogenase A (LDHA), thereby promoting the progression of HCC (Wang et al., 2022a). Zhang et al. (2018) found that hypoxic stress in HCC cells can promote the binding of YAP to HIF-1α in the nucleus, maintain the stability of HIF-1α, and activate pyruvate kinase M2 (PKM2) transcription to accelerate glycolysis. In addition, as upstream targets of PKM2, AKT, CAMKβ, and GTPBP4 can also affect the proliferation and glycolysis of HCC cells by regulating PKM2 (Ye et al., 2019; Sheng et al., 2020; Zhou et al., 2022). Similar to oncogenic molecules, Akt can also promote metabolic reprogramming by increasing GLUTs expression (Hu et al., 2019). These studies highlight the role of glucose metabolism in the development and progression of HCC, making glucose metabolism a potential target for the treatment of this disease.

Evidence has indicated that many traditional Chinese herbal medicines (TCM) exhibit potential anti-cancer effects (Liu et al., 2019; Fan et al., 2020; Li et al., 2022a). However, the underlying molecular mechanisms and targets remain unclear. Thus, to improve their development as new anticancer drugs, the pharmacological effects of TCM preparations must be thoroughly assessed (Chen and Ye, 2022). Schisantherin A (STA) is a TCM extracted from the plant *Schisandra chinensis*, which has a wide range of pharmacological properties, including anti-inflammatory and anti-oxidant effects (Gong and Wang, 2018; Gui et al., 2020; Wang et al., 2021). STA has also been used in the treatment of cancer by inducing cell apoptosis and inhibiting cell proliferation. Wang et al. (2020) reported that STA could induce apoptosis and cell cycle arrest *via* the production of reactive oxygen species and activation of JNK signaling with Nrf2 inhibition in gastric cancer cells. However, the anti-HCC effect of STA or its molecular mechanisms were not examined. Thus, an accurate understanding of the biological functions and mechanisms of STA can provide new insights for the treatment of HCC.

This study assessed the anti-tumor effects of STA against liver cancer as well as the underlying mechanisms. The significant inhibitory effect of STA on the migration and proliferation of HCC cells was observed. Further, glucose metabolism inhibition was shown to be involved in STA-induced anti-proliferation and migration. In addition, a subcutaneous tumor model in nude mice confirmed that STA significantly inhibited the growth of solid tumors. Thus, STA may be a new drug for the treatment of liver cancer.

## 2 Materials and methods

### 2.1 Cell culture

Human hepatoma cell lines, including highly metastatic HCCLM3 and low-metastatic Hep3B, were obtained from Liver Cancer Institute, Zhongshan Hospital, Fudan University (Shanghai, China). The cells were cultured in DMEM (GIBCO, United States) containing 10% fetal bovine serum (FBS, Gibco, United States) and 100 U/mL penicillin/streptomycin (GIBCO, United States), and maintained at 37°C with 5% CO_2_. STA (purity ≥ 98%; MedChemexpress, New Jersey, United States) was dissolved in dimethyl sulfoxide (DMSO; Solarbio, Beijing, China) to obtain a 20-mM stock solution that was then diluted with DMEM.

### 2.2 Cell viability analysis

For cytotoxicity assays, 5,000 cells per well were seeded in triplicate in 96-well plates and incubated at 37°C and 5% CO_2_ for 24 h. Cells were treated with different doses of STA for 48 h as required. Cell viability was measured with a cell counting kit (CCK-8; Dojindo, Japan) according to the manufacturer’s instructions. CCK-8 reagent (10 μl) was mixed with cells and incubated for 1 h. The final absorbance at 450 nm was assessed in each well using a MultiSkan GO micropore meter (Thermo, United States).

### 2.3 5-Ethynyl-2′-deoxyuridine assay

Based on the results of the cell viability analysis, three groups of cells (DMSO, 10 μM, 30 μM, and 50 μM) were seeded in 96-well plates at 5,000 cells/well for 48 h with 1.5 μM cisplatin (CDDP) administered as a positive control. HCC cells were then incubated with 50 μM 5-ethynyl-2′-deoxyuridine reagent (EdU; RiboBio, Guangzhou, China) for 2 h for EdU incorporation during DNA synthesis. Cells were then stained with Apollo fluorescent dye for 1 h and 0.25% Triton X-100 was added to each well. Finally, the samples were stained with Hoechst 33,342. Fluorescence images were captured under a fluorescence microscope (Olympus, Tokyo, Japan). The EdU assay was performed three times using five biological replicates.

### 2.4 Western blotting

Proteins were analyzed by western blotting according to standard methods. Proteins were extracted from cells using RIPA buffer (Beyotime, China) containing a mixture of protease and phosphatase inhibitors and quantified using an Enhanced BCA Protein Assay Kit (Beyotime, China). Protein samples (30  μg/lane) were loaded onto 10% separating gel and blotted onto a PVDF membrane (Millipore, Billerica, MA, United States). After blocking with 5% skimmed milk powder for 1 h, the membranes were incubated overnight with the following primary antibodies at 4°C: anti-CDK1(Zen-BioScience, China), anti-survivin (Zen-BioScience, China), anti-MMP2 (Zen-BioScience, China) and anti-GAPDH (Zen-BioScience, China). Membranes were then washed and incubated with HRP-linked secondary antibody for 1 h at 37°C. Finally, the samples from three independent experiments were detected using the ECL reagent (Thermo Fisher, United States).

### 2.5 Clone formation assay

Clone formation was evaluated on Hep3B and HCCLM3 cells in response to STA treatment (DMSO 10 μM, 30 μM, and 50 μM). Cells in the logarithmic growth phase were seeded at 500 cells/well in six-well plates and cultured at 37°C and 5% CO_2_ for 5 days. After treatment with fresh media containing different concentrations of STA for 5 days, the cells were fixed with 4% paraformaldehyde (PFA) at 4°C overnight. Finally, after staining with 0.1% crystal violet, cell colonies were photographed and counted.

### 2.6 Wound healing assay

Hep3B and HCCLM3 cells were seeded in six-well plates and allowed to grow to ∼70% density. A sterile 200-μl pipette tip was then used to scratch the surface, fresh medium containing different concentrations of STA (DMSO 10 μM, 30 μM, and 50 μM) was replaced, and the cells were cultured for 48 h. After fixing with 4% PFA, photographs were taken after staining with crystal violet to estimate the area occupied by migrating cells.

### 2.7 RNA sequencing and data analysia

Following treatment with STA for 48 h, total RNA was extracted from Hep3B and HCCLM3 cells using TRIzol reagent (Invitgen, Thermo Scientific, United States). Partially isolated RNA samples were submitted to BGI Co., Ltd., (Shenzhen, China) for transcriptome sequencing and BGISEQ-500 analysis.

### 2.8 Metabolite extraction and gas chromatography-mass spectrometric analysis

Hep3B cells were cultured in a 10-cm petri dish and treated with 50 μM STA for 48 h. The cells were washed twice with PBS solution and then peeled off by cell scraping. After centrifugation, the supernatant was removed, and the precipitated cells were collected and flash-frozen in liquid nitrogen for 15 min. Metabolites were extracted from equal amounts of STA-treated or untreated Hep3B cells (about 1 × 10^7^ cells) by the previously mentioned method (Qu et al., 2022). The Trace1300 gas chromatography-mass spectrometry analysis system (Thermo Scientific) was used for metabolomics analysis in conjunction with the TCM Chromatographic Fingerprint Computer-Aided Similarity Assessment System (2012 edition) and SIMCA software (Umetrics, Sweden). An Agilent DB-5MS chromatographic column was used with an injection volume of 1 μl. The ion source of mass spectrometry was electrospray ionization (ESI), using the positive ion mode. The inlet temperature was 280°C. The control group was treated with DMSO; eight samples were collected for each group.

### 2.9 Animal studies

Female nude mice (*n* = 24) obtained from the Laboratory Animal Center of Chongqing Medical University were used for animal experiments. For subcutaneous xenograft experiments, 2 × 10^6^ Hep3B cells in the logarithmic growth phase were injected into the armpit of nude mice. After 12 days, the mice were randomly divided into a control group, a positive control group (CDDP, 5 mg/kg, i.p., once in 2 days), a low concentration group (STA, 10 mg/kg i.p., once in 2 days), and a high concentration group (STA, 20 mg/kg i.p., once in 2 days), with six mice in each group. Intraperitoneal injections were performed every 2 days, and the longest axes (L) and vertical axes (R) of the tumor were measured. The tumor volume (V) was calculated according to V = (π/6) × L × *R* ^2^. Sixteen days later, the tumor was excised for weighing and histological analysis.

### 2.10 Histological analysis

For hematoxylin and eosin (H&E) staining, all grafts were fixed in 4% PFA overnight. After dehydration, the samples were embedded in paraffin, sectioned, and stained with H&E. For immunohistochemistry detection, PFA-fixed tissues were immunostained for Ki67 protein using standard immunohistochemical procedures according to the manufacturers’ instructions and incubated overnight. After washing three times with PBS, the cells were incubated with secondary antibody at 37°C for 30 min and stained with DAB (3,3′-diaminobenzidine); Ki67-positive cells were brown.

### 2.11 Statistical analysis

Data were expressed as mean ± SEM of at least three independent experiments. Two-tailed Student *t*-tests were used to calculate the *p* values; *p* < 0.05 was considered statistically significant.

## 3 Results

### 3.1 STA inhibits the survival of HCC cells

**FIGURE 1:**
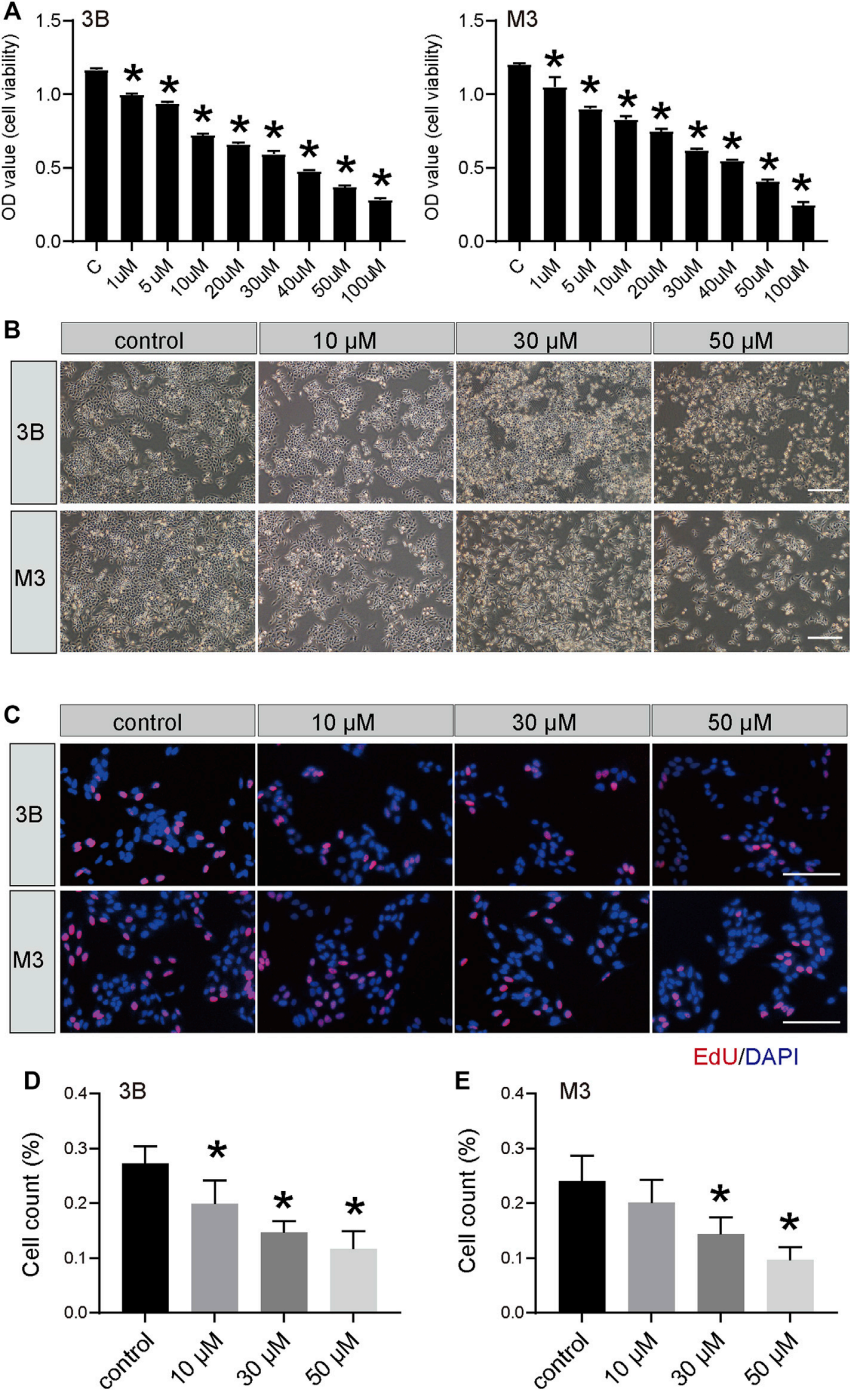
STA inhibits the survival of HCC cells. **(A)** Cell viability of Hep3B and HCCLM3 cells treated with STA at indicate concentration was determined by CCK8 method, DMSO was used as control. **(B)** The morphology of Hep3B and HCCLM3 cells treating with STA in different concentration for 48 h. **(C–E)** EdU-positive Hep3B and HCCLM3 cells after treating with STA for 48 h. Scale bar = 100 μm. Data shown are means ± SD; *n* = 3. *p < 0.05, significantly from control group.

To determine the effect of STA on cell viability, Hep3B and HCCLM3 cells were treated with different concentrations of STA for 48 h and detected by CCK-8 assay. STA decreased the viability of Hep3B and HCCLM3 cells in a dose-dependent manner (Figures 1A,B); at 30 μM and 50 μM, STA significantly inhibited the viability of Hep3B and HCCLM3 cells, whereas at 100 μM, most cells had died. Furthermore, the number of exfoliated cells increased and the cell size shrank with the increase in STA concentration (Figure 1C). Therefore, concentrations of 10 μM, 30 μM, and 50 μM STA were selected for subsequent experiments.

### 3.2 STA inhibits Hep3B and HCCLM3 cell proliferation and migration

**FIGURE 2:**
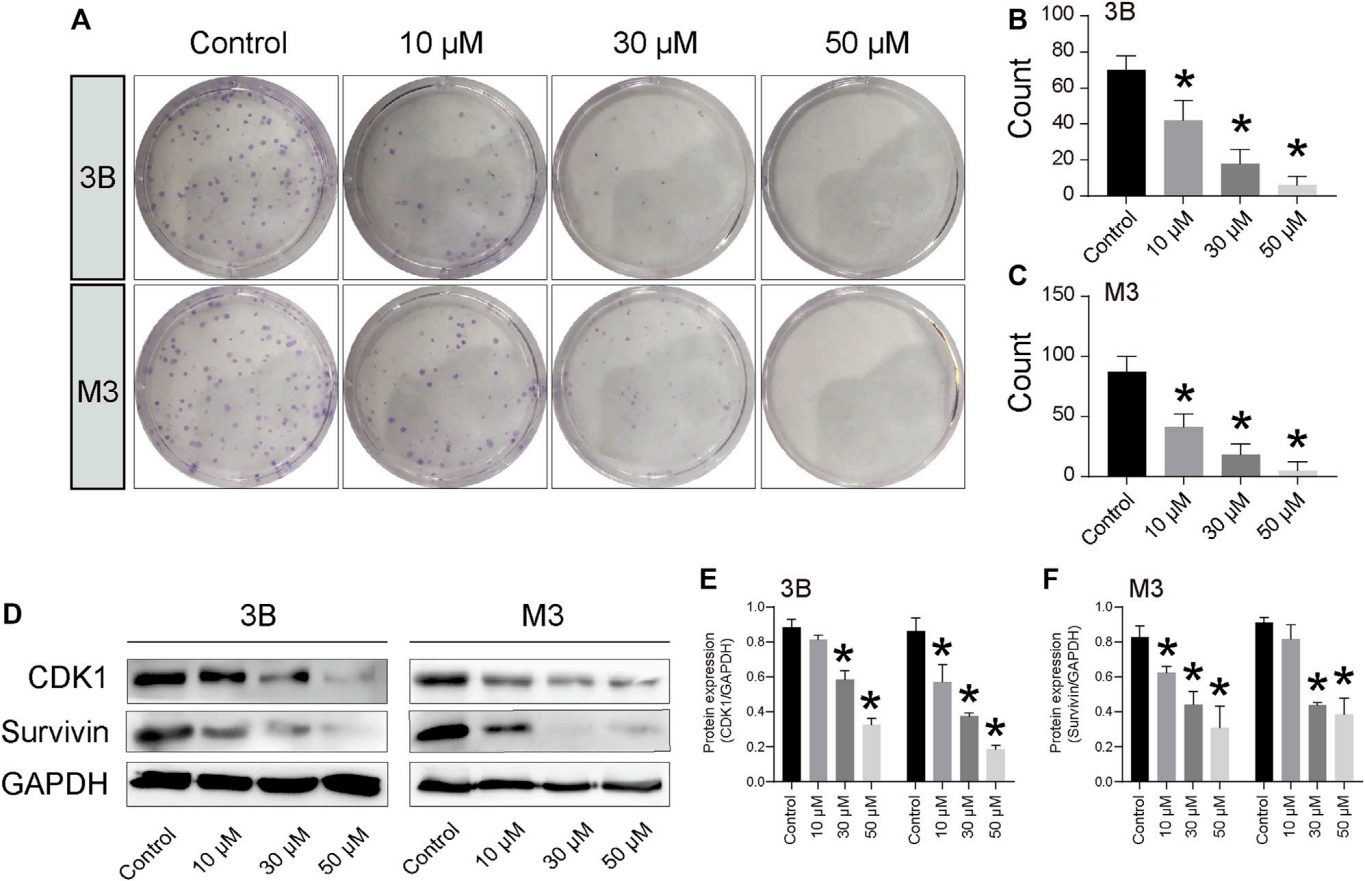
STA inhibits Hep3B and HCCLM3 cell proliferation. **(A–C)** Clone formation of Hep3B and HCCLM3 cells treated with STA at 10 μM, 30 μM, and 50 μM. **(D–F)** The representative bands of CDK1 and survivin in Hep3B and HCCLM3 cells treated with STA at 10 μM, 30 μM, and 50 μM for 48 h. GAPDH was used as an internal control. Data shown are means ± SD; *n* = 3. *p < 0.05, significantly from control group.

The EdU and clone formation assays were used to evaluate the effect of STA on cell proliferation. EdU showed that, compared with the control group, DNA synthesis and the EdU positivity rate were significantly decreased in cells treated with STA for 48 h in a dose-dependent manner (Figures 1D,E). Next, the effect of STA on the self-renewal ability of HCC cells was observed by clone formation assay. The number and size of clones in the STA-treated groups were significantly reduced compared with the control group (Figures 2A,B). The expression of proliferation-related proteins was further examined by western blotting assay, which demonstrated that the expression of CDK1 and survivin proteins was significantly decreased (Figures 2C–F). Therefore, STA effectively inhibited the proliferation of HCC cells.

**FIGURE 3:**
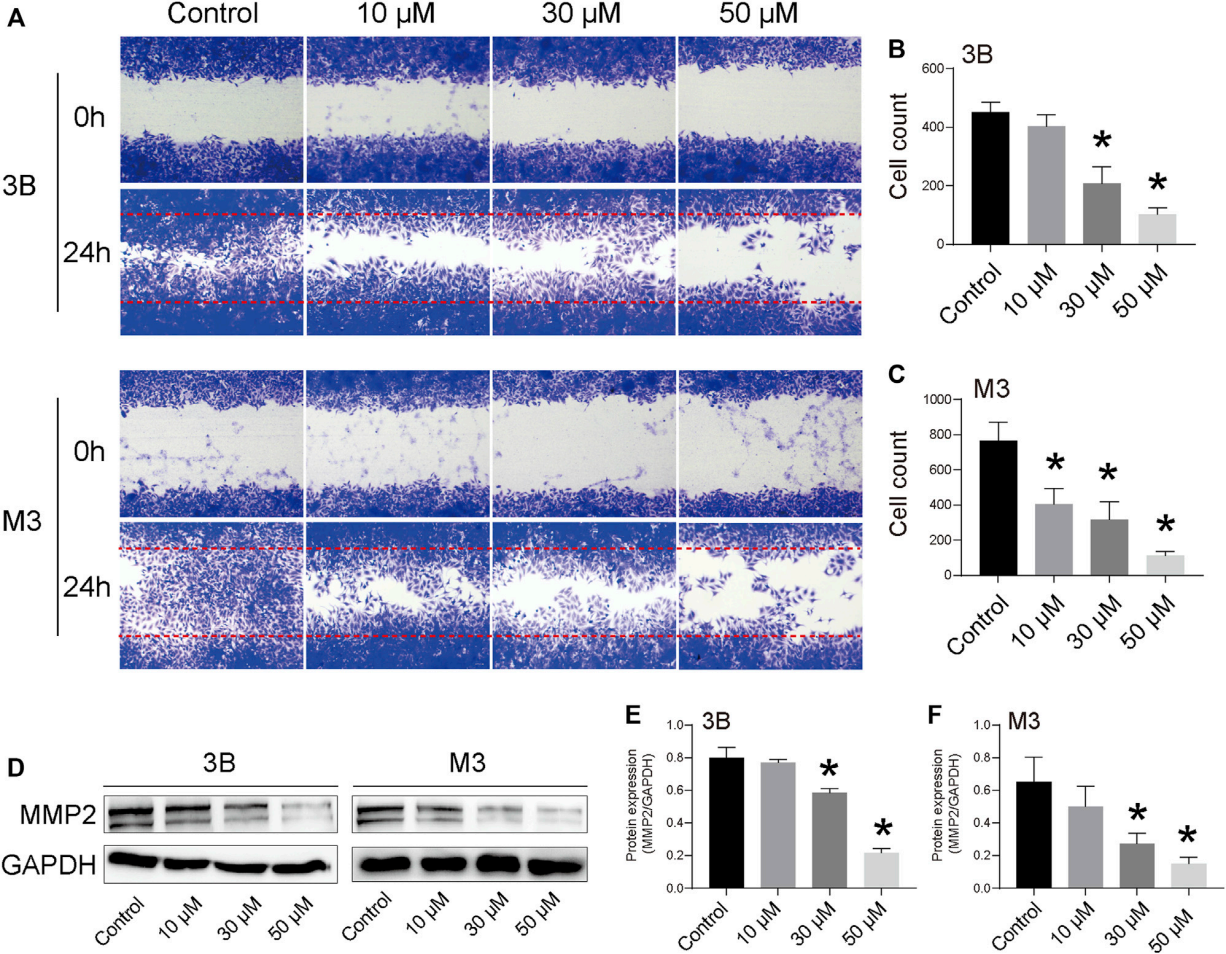
STA inhibits Hep3B and HCCLM3 cell migration. **(A)** Scratch assay was performed to determine migration of Hep3B and HCCLM3 cells treated with STA at for 48 h. **(B,C)** Quantification of the scratch image was performed by calculating the migrated cell counts in **(A)**. **(D–F)** The representative bands of MMP2 in Hep3B and HCCLM3 cells treated with STA for 48 h. GAPDH was used as an internal control. Data shown are means ± SD; *n* = 3. *p < 0.05, significantly from control group.

The cell scratch assay revealed that STA significantly inhibited the migration of Hep3B and HCCLM3 cells (Figures 3A–C). After 48 h, cells in the control group almost completely filled the scratch area, whereas the number of cells migrating to the scratch area was significantly reduced with the increase in STA concentration. Furthermore, STA treatment inhibited the expression of MMP2, a migration-related protein (Figures 3D–F).

### 3.3 STA regulates differentially expressed genes and pathway enrichment analysis

**FIGURE 4:**
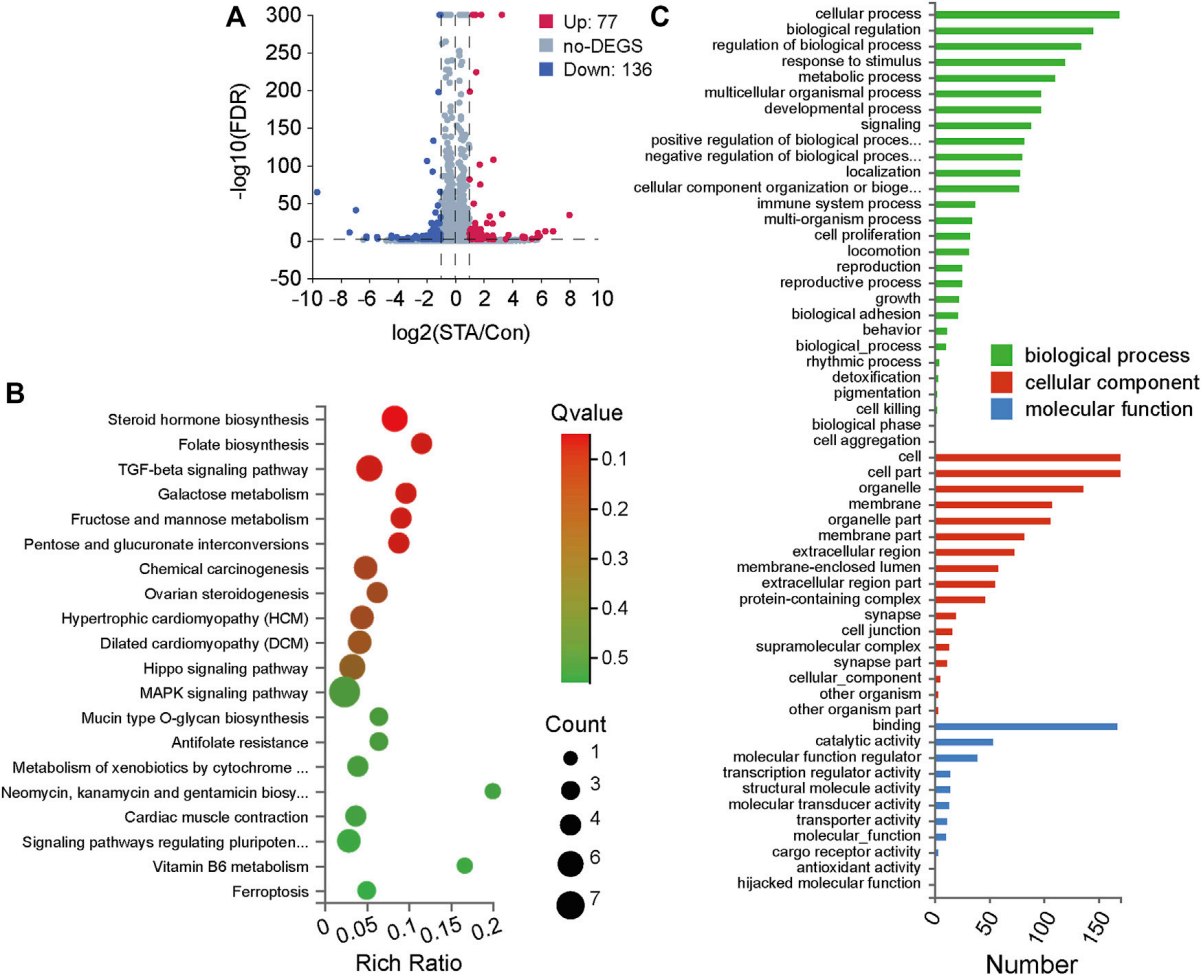
STA regulates the differentially expressed genes and pathway enrichment analysis in Hep3B cells. **(A)** Significant differentially expressed genes were shown in volcano plot. FC (fold change) > 1 was accepted as positive differentially expressed genes, up for 77; down for 136. **(B)** KEGG pathway enrichment analysis, a larger *p*-value (−Log10) shows a higher degree of enrichment. **(C)** GO annotations analysis of Hep3B cells treated with STA, compared with control group.

To investigate how STA regulates cell growth and proliferation, transcriptome analyses on cells treated with 30 μM STA and DMSO were performed. The global gene expression profile showed that STA regulated gene expression in Hep3B cells. Compared with the control group, 77 genes were upregulated and 136 genes were downregulated in the STA-treated group. Thus, STA significantly inhibited the transcription of genes, leading to the arrest of basic cell functions (Figure 4A). Subsequently, KEGG pathway enrichment analysis of differentially expressed genes showed that STA affected glucose metabolism of HCC cells, specifically galactose metabolism and fructose and pentose phosphate metabolism (Figure 4B). Gene ontology functional annotation also showed that STA was involved in the regulation of metabolic processes (Figure 4C).

### 3.4 STA affects the glucose metabolism pathway of Hep3B cells

**FIGURE 5:**
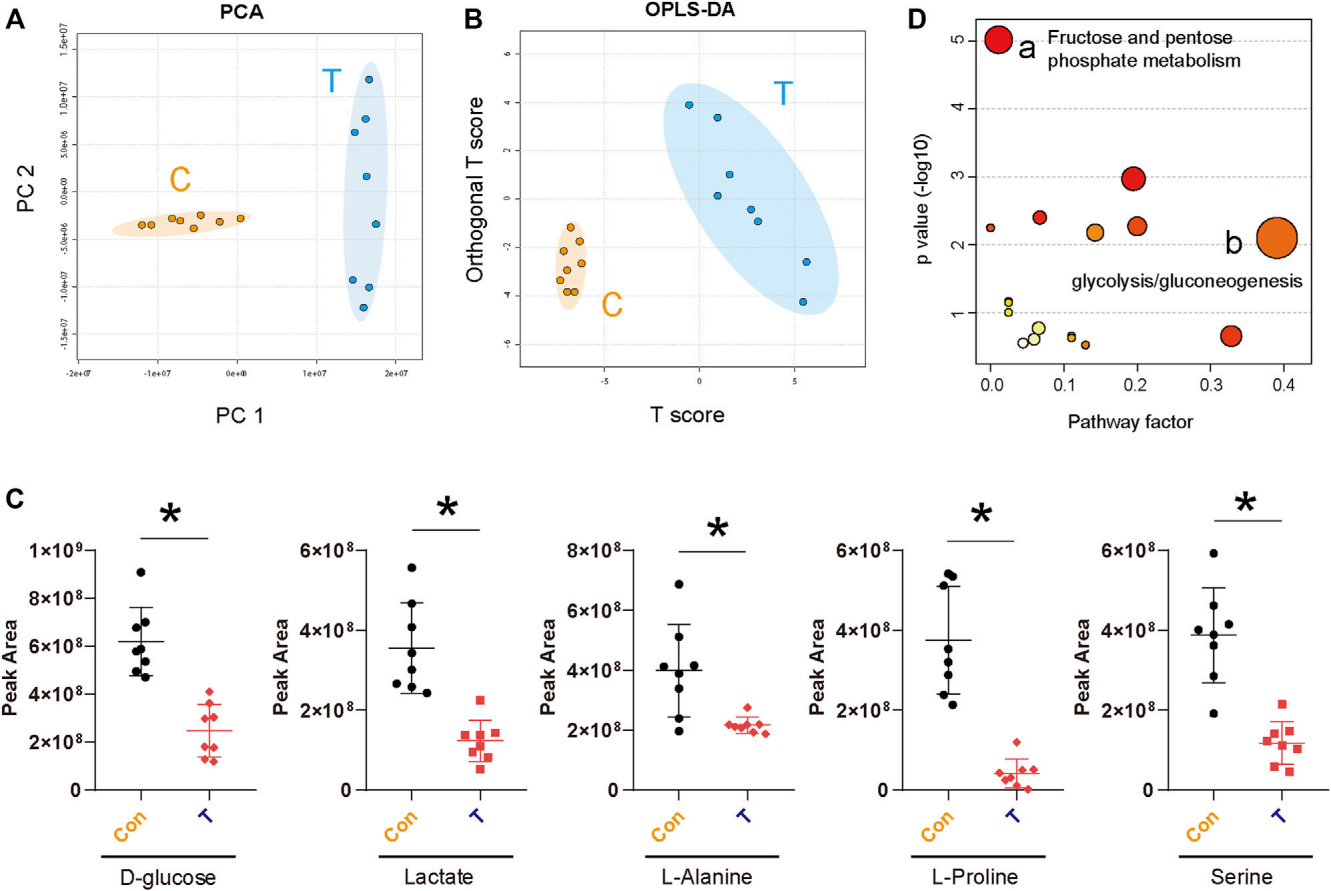
STA affects the glucose metabolism pathway of Hep3B cells. **(A)** The PCA score plot of Control and Drug group (30 μM), it represents samples in the groups were closely cluster to one another. **(B)** The OPLS-DA score plot of Control and Drug group revealed the clustering of samples in the training set. **(C)** Metabolites altered by STA treatment in Hep3B cells. *n* = 8 replicates per group. **(D)** Pathway enrichment of differential metabolites. Mainly enriched in fructose and pentose phosphate metabolism and glycolysis/gluconeogenesis.

Metabolic reprogramming is an important feature of malignant tumors (Du et al., 2022; Liao et al., 2022). In cancer, malignant tumor cells respond to a variety of endogenous and exogenous cues to obtain metabolic adaptation, promoting the growth of tumor cells (Faubert et al., 2020). Previous KEGG pathway enrichment analyses showed that STA treatment could regulate glucose metabolism in Hep3B cells (Figure 4). Therefore, metabolomics was used for further validation analyses. GC-MS was used to explore the effect of STA on the production of primary metabolites in HCC cells. Principal component analysis (PCA) showed that metabolites induced by DMSO and STA (30 μM) were tightly clustered (Figure 5A). The difference between DMSO and STA (30 μM) was significant using OP-LSDA analysis to exclude non-significant variables and to evaluate only statistically significant signals (Figure 5B). STA treatment significantly downregulated the production of D-glucose, lactate, L-alanine, L-proline, and serine (Figure 5C). Furthermore, the differential metabolites were mainly enriched in fructose and pentose phosphate metabolism and glycolysis/gluconeogenesis (Figure 5D), suggesting that the antitumor effects of STA are mediated by the regulation of glucose metabolism.

### 3.5 STA impairs growth of Hep3B cells *in vivo*

**FIGURE 6:**
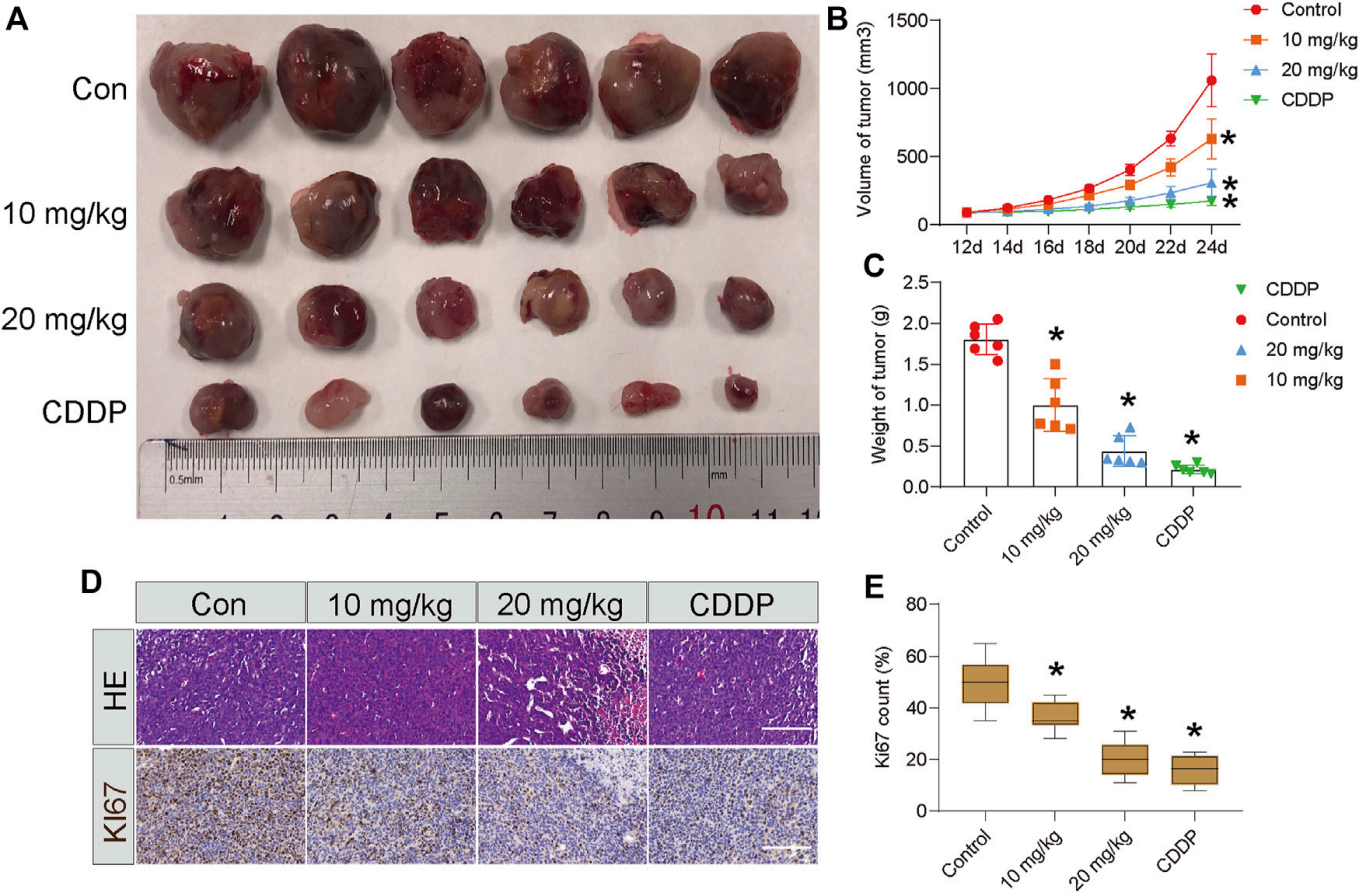
STA impairs the growth of Hep3B cells *in vivo*. Twenty-four nude mice were randomly divided into negative control group, positive control group (CDDP, 5 mg/kg, i.p., once two days), low-concentration treatment group (STA, 10 mg/kg, i.p., once two days) and high-concentration treatment group (STA, 20 mg/kg, i.p., once two days). **(A)** Photograph of tumors from indicated mice. **(B)** Tumor volume of indicated mice. **(C)** Tumor weight of indicated mice. **(D)** H&E staining and IHC of KI67 in indicated tumors. **(E)** Quantitative image analysis of KI67 in **(D)**. Scale bar = 50 μm. All data were analyzed using unpaired Student *t*-Tests and are shown as the means ± SD. *p < 0.05.

To assess the antitumor effect of STA *in vivo*, Hep3B cells were subcutaneously injected into the axilla of 6-week-old nude mice and allowed to grow for 10 days. After 12 days of treatment, both STA and CDDP significantly reduced tumor weight and volume compared with the control group (Figures 6A–C). H&E staining of tumor samples showed that the number of cells in the STA-treated group was significantly lower than that in the control group. Furthermore, the expression of Ki67 decreased significantly with the increase in STA concentration (Figures 6D,E). Thus, STA had a significant inhibitory effect on tumor growth *in vivo*.

## 4 Discussion

Globally, liver cancer is one of the most common fatal malignancies and is the fifth most common malignancy in China (Pan et al., 2022). Patients are commonly diagnosed at an advanced stage, leading to poor prognosis (Chen et al., 2020). Chemotherapy is the most accessible treatment option for patients with liver cancer (Sacco et al., 2017); however, only a small proportion of patients benefit from treatment, with considerable resistance within 6 months after the start of the treatment regimen (El-Serag et al., 2008). Furthermore, the long-term use of chemotherapy drugs (e.g., sorafenib) leads to toxicity and/or drug ineffectiveness (Colagrande et al., 2015; Tang et al., 2020). Therefore, new treatment options are urgently required for the treatment of liver cancer. Growing evidence has indicated that active ingredients in Chinese herbal medicines play a role in inhibiting mechanisms involved in the development of cancer, with stimulating mechanisms associated with disease prevention (Lam et al., 2022). These compounds have been shown to exert anticancer (Zhao et al., 2021), anti-inflammatory (Tao et al., 2022), and anti-oxidant effects (Gu et al., 2022), and may provide new treatment options for cancer.

As a monomeric TCM compound, STA retrieved form *S. chinensis* (Turcz.) Baill has multiple pharmacological activities, including anti-oxidant and anti-inflammatory activities, both *in vivo* and *in vitro* (Szopa et al., 2018; Fu et al., 2021). STA has been reported to mitigate liver injury induced by lipopolysaccharide, IL-1β, and cigarette smoke extract (Jiang et al., 2015). Indeed, chronic inflammation (Thadathil et al., 2022), smoking (Wen et al., 2022), and fatty liver disease (Paternostro and Trauner, 2022) are the main causes of liver cancer. Previous studies showed that STA can inhibit the proliferation of human hepatic stellate cells, leading to the speculation that STA might have an inhibitory effect on liver cancer. Furthermore, STA has been shown to inhibit the proliferation and migration of gastric cancer through the production of reactive oxygen species and JNK signaling pathway activation (Wang et al., 2020). Therefore, it was hypothesized that STA would have antiproliferative effects on liver cancer cells and inhibit cell clone formation and migration through inhibition of the related molecular mechanisms.

Herein, STA significantly suppressed cell proliferation and migration, and reduced the viability of liver cancer cells *in vitro* and *in vivo*. STA displayed potent inhibitory effects on proliferation by decreasing the expression of CDK1 and survivin. In addition, STA inhibited cell migration through the downregulation of MMP2 expression. Further transcriptome sequencing and KEGG signaling pathway enrichment data showed that the metabolic process in Hep3B cells treated with STA had changed. Growing evidence suggests that metabolic disorder is a significant hallmark of malignant tumors that controls key tumor biological processes such as the proliferation, migration, and invasion of tumor cells (Dai et al., 2022; Sun et al., 2022; Tan et al., 2022). Thus, by inhibiting the metabolic pathways of tumor cells, the occurrence and development of tumors could be prevented. Therefore, targeting tumor metabolism is a promising therapeutic approach (Wang et al., 2022b; DeBerardinis and Keshari, 2022; Reyes-Castellanos and Carrier, 2022). Metabonomic results indicated that STA treatment decreased the production of D-glucose, lactate, L-alanine, L-proline, and serine. Both lactate and serine have been reported to be associated with the development and exacerbation of tumors (Woo et al., 2016; Bogdanov et al., 2022). Herein, these differential metabolites were mainly enriched in fructose and pentose phosphate metabolism and glycolysis/gluconeogenesis. Our results suggest that STA can inhibit glucose metabolism in Hep3B cells.

Studies have shown that glucose metabolism disorder is a representative metabolic feature in HCC (Tsujimoto et al., 2022). An increase in the degree of glycolysis in tumors occurs to meet the energy demands of rapidly proliferating tumor cells, and glycolysis can also produce intermediate metabolites supporting the biosynthesis of nucleotides, amino acids, and lipids, thus allowing the rapid proliferation of tumor cells (Feng et al., 2020). Glucose metabolism is coupled to cell cycle progression, and glucose metabolism ensures adequate ATP and synthetic metabolites at different stages of the cell cycle (Cheung et al., 2022). 6-Phosphofructo-2-kinase (PFKFB3) can promote cell cycle progression through CDK1-mediated phosphorylation of P27 (Yalcin et al., 2014). In turn, the cell cycle is also involved in the regulation of glucose metabolism. Tang et al. revealed that CDK2 can increase glycolysis by inhibiting SIRT 5 in gastric cancer (Tang et al., 2018). High expression of survivin can shut down mitochondrial complex 1 activity, thereby switching neuroblastoma cells from OXPHOS to glycolysis (Hagenbuchner et al., 2016). Our western blotting results also showed that the protein expression of CDK1 and survivin was decreased in STA-treated HCC cells (Figure 2D). In addition, enhanced glycolysis promotes the conversion of pyruvate to lactate catalyzed by LDHA. Studies have confirmed that LDHA levels in tissues and plasma are significantly increased in patients with liver cancer, and increased LDHA levels are significantly associated with poor prognosis (Faloppi et al., 2016; Guo et al., 2019). Our results showed that STA could reduce lactate production in Hep3B cells (Figure 5C). Increased lactate production can induce a more acidic tumor microenvironment, which is conducive to tumor metastasis, angiogenesis, and immunosuppression (Li et al., 2022b). Angiogenesis is conducive to the delivery of oxygen and nutrients to tumor tissues, and meets the needs of rapid proliferation of tumor cells (Qi et al., 2022).

Furthermore, *in vivo* subcutaneous tumor-formation experiments in nude mice confirmed that STA could significantly inhibit the growth of solid tumors. Immunohistochemistry showed that the anti-tumor effect of STA was related to the inhibition of expression of Ki67, a marker of tumor proliferation (Figure 6D).

## 5 Conclusion

In summary, our findings suggest that STA treatment inhibits cell proliferation and migration by regulating glucose metabolism in HCC cells. The clone formation assay and cell scratch assay indicated that STA treatment reduced the proliferation and migration capacity of Hep3B and HCCLM3 cells *in vitro*. Further, transcriptome sequencing and metabolomics results showed that STA treatment could regulate glucose metabolism and reduce D-glucose and lactate production in Hep3B cells. The subcutaneous tumorigenesis assay in nude mice further verified that STA treatment inhibits the growth of HCC tumors *in vivo*. Based on our results, STA has the potential to be developed as a therapeutic agent for the treatment of liver cancer.

## Data Availability

The datasets presented in this study can be found in online repositories. The names of the repository/repositories and accession number(s) can be found in the following: NCBI BioProject PRJNA869496.

## Abbreviations

CCK-8, cell counting kit; CDDP, cisplatin; DMSO, dimethyl sulfoxide; EDU, 5-Ethynyl-2′-deoxyuridine; FBS, fetal bovine serum; GLUTs, glucose transporters; HCC, hepatocellular carcinoma; H&E, hematoxylin and eosin; OXPHOS, oxidative phosphorylation; LDH, lactate dehydrogenase; PFKFB3, 6-Phosphofructo-2-kinase; PKM2, pyruvate kinase M2; TCM, traditional Chinese herbal medicines; STA, Schisantherin A.
